# Added Value of Assessing Adnexal Masses with Advanced MRI Techniques

**DOI:** 10.1155/2015/785206

**Published:** 2015-08-27

**Authors:** I. Thomassin-Naggara, D. Balvay, A. Rockall, M. F. Carette, M. Ballester, E. Darai, M. Bazot

**Affiliations:** ^1^INSERM, UMR970, Team 2, Angiogenesis Imaging, 75005 Paris, France; ^2^AP-HP, Hôpital Tenon, Department of Radiology, 58 avenue Gambetta, 75020 Paris, France; ^3^Sorbonne University, UPMC Univ Paris 06, IUC, 75005 Paris, France; ^4^Imperial College London, ICTEM Building, Room 136, 1st Floor, Du Cane Road, London W12 0NN, UK; ^5^AP-HP, Hôpital Tenon, Department of Gynaecology and Obstetrics, 4 rue de la Chine, 75020 Paris, France

## Abstract

This review will present the added value of perfusion and diffusion MR sequences to characterize adnexal masses. These two functional MR techniques are readily available in routine clinical practice. We will describe the acquisition parameters and a method of analysis to optimize their added value compared with conventional images. We will then propose a model of interpretation that combines the anatomical and morphological information from conventional MRI sequences with the functional information provided by perfusion and diffusion weighted sequences.

## 1. Introduction

The clinical suspicion of an adnexal mass is one of the most frequent indications for gynecologic imaging. In this setting, the first imaging technique is ultrasonography with Doppler [1–6]. A large majority of women will not need any other imaging technique for characterization of the adnexal lesion because the lesion either has typically benign features (such as anechoic cyst) or is overtly malignant (such as the presence of peritoneal implants) [7]. In the latter case, the extent of disease will then be determined with computed tomography (CT) which is the current standard of care for preoperative staging.

However, when an echoic area is discovered at ultrasound, the question remains whether or not this represents a solid soft tissue component consistent with tumour. If the sonographer detects blood flow within the echoic area, a solid component surely exists but many benign lesions may display a solid component [8]. If no blood flow is detected in the echoic portion, the question remains without answer as solid tissue may not display any blood flow on ultrasound [9]. Thus, when no typical signs of benignity or malignancy are present, the lesion remains indeterminate and a second line technique is needed with a frequency that depends on sonographer's experience [10, 11].

Pelvic magnetic resonance (MR) imaging has clearly been demonstrated to be the best imaging technique to characterize indeterminate or complex adnexal masses due to its excellent tissue contrast [12, 13]. Firstly, the conventional sequences (T2, T1, and T1 with fat saturation) are evaluated. MR imaging is very accurate for the identification of endometriotic or fatty masses using these sequences whereas sonography can only suggest these diagnoses in some cases [14, 15]. Moreover, conventional MR imaging readily distinguishes cystic from solid soft tissue components, as solid soft tissue enhances after gadolinium injection [16, 17]. Malignancy can only be suggested if there are enhancing solid components, such as thickened irregular septa, solid papillary projections, or a solid mass. These enhancing solid components are grouped under the name of “solid tissue” [18, 19]. Others types of solid component such as thin smooth septa or cyst wall are not considered as solid tissue and do not require any functional characterization. If solid tissue is detected within an adnexal lesion, early publications demonstrated that the T2 signal intensity of the solid tissue is useful to distinguish benign from malignant tumors [12] because some benign tumors contain fibrous material in their solid tissue and thus appear with a typically low T2 weighted signal. The accuracy of MRI interpretation to differentiate benign from malignant masses using only the conventional sequences is about 80% according to the published literature [17, 20–22]. However, Huchon et al. demonstrated that most ovarian tumors undergo surgery without any MR analysis and there is a high rate of incomplete surgery [23]. More recently, perfusion and diffusion weighted sequences were demonstrated to improve diagnostic confidence about 25% and 15%, respectively [24], allowing an accuracy of up to 94.6%. This increase in the diagnostic accuracy of MRI may help to increase the clinician's confidence in MR imaging.

This review will present these two functional techniques that are readily available in clinical routine, including acquisition parameters, method of analysis, and added value compared with conventional images. Then, we will propose a synthesis consisting of an interpretation model combining conventional and functional criteria.

## 2. Perfusion Weighted MR Sequence

Ovarian cancer is characterized by an anarchic neovascularization resulting in a wide number of immature microvessels. These vessels are characterized by a lack of coverage by pericytes and the higher expression of one of the receptors of Vascular Endothelial Growth Factor (VEGF) named VEGFR-2 on both endothelial and epithelial cells of ovarian cystadenocarcinomas. These physiopathogenic characteristics have been demonstrated to be in line with variations of MR perfusion parameters [25].

### 2.1. Technical Features

All the parameters required to perform MRI for adnexal masses characterization are provided in Table 1.

### 2.2. MR Acquisition Parameters

**Figure 1:**
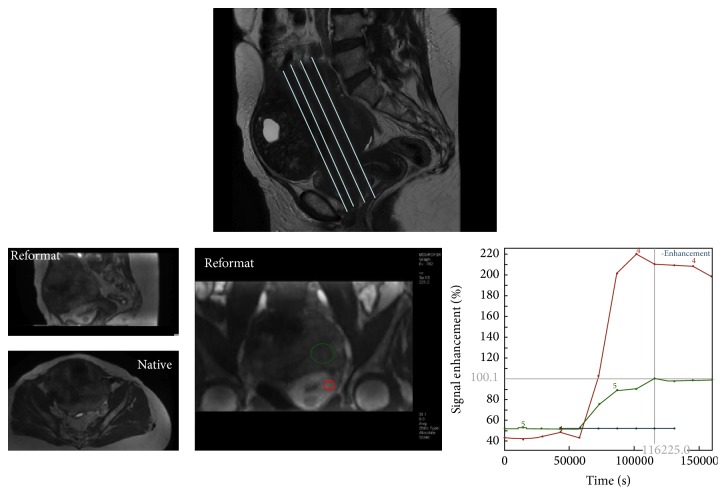
Perfusion MR acquisition. The sequence may be acquired in 2D plane in order to cover both external myometrium and the tumor or in axial 3D plane with a high quality of reformatting imaging in sagittal and coronal planes.

For female pelvic imaging, perfusion MR technique is based on a Dynamic Contrast Enhanced (DCE) Gradient Echo (GRE) T1 weighted sequence. The main important parameters include a temporal resolution which must be lower than 15 seconds and the total sequence duration should be at least 3 minutes. External myometrium is used as an internal reference because it is enhanced to approximately the same extent as ovarian tumour tissue [26]. Ideally, an axial 3D GRE T1 sequence should be performed with high quality reformats postcontrast in the sagittal and coronal planes. If only a 2D sequence can be performed, the acquisition plane should be placed in order to cover external myometrium and the tumor, both of which need to be analyzed [27] (Figure 1).

### 2.3. MR Analysis

**Figure 2:**
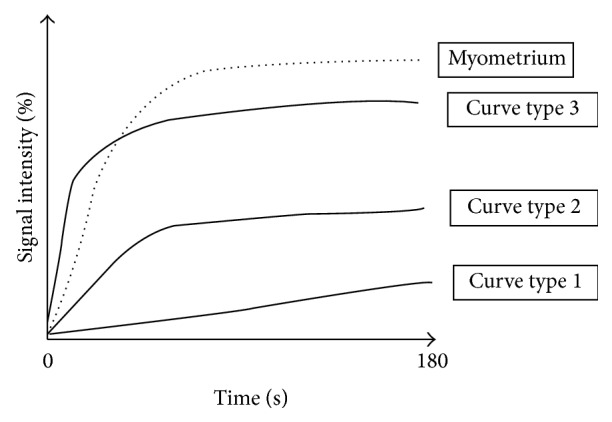
Time intensity curve.

For perfusion data, three types of analysis exist. The first one is the time intensity curve analysis. For adnexal mass characterization, as shown in Figure 1, two regions of interest are placed on both external myometrium and solid tissue of the adnexal mass on DCE MR sequence. Then, the evolution of relative signal intensity according to time can be assessed using time intensity curves. For characterization of adnexal masses, time intensity curve of solid tissue in an adnexal mass is compared to that of external myometrium, which acts as the internal reference. The use of an internal reference overcomes to some extent the lack of reproducibility which is well known to be the main limitation of this type of postcontrast perfusion data. Thus, when solid tissue is enhanced with a weak and progressive curve in comparison to the myometrium, the curve is named “type 1.” When solid tissue is enhanced with a moderate enhancement in comparison to myometrium with a plateau, the curve is named “type 2.” Finally, when solid tissue is enhanced with a curve steeper than that of myometrium, whatever the intensity of enhancement, the curve is named “type 3” [28] (Figure 2).

**Figure 3:**
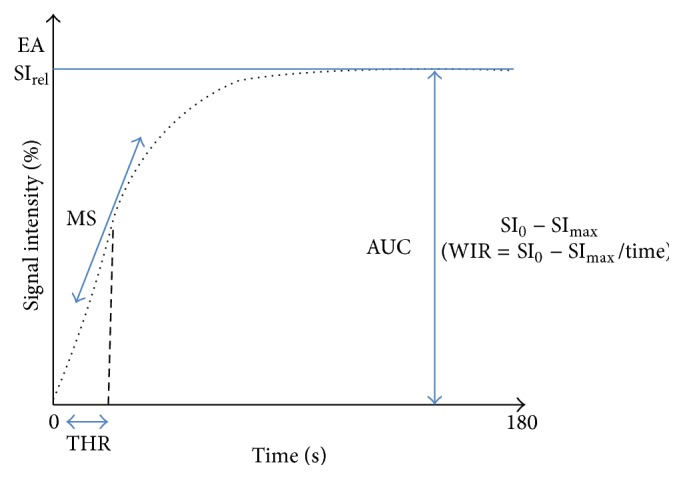
Semiquantitative analysis. Different parameters published were EA (enhancement amplitude), THR (time of half rising), maximal slope (MS), SI_rel_ (maximal relative enhancement), WIR (wash-in rate), and SI_max_.

The second type of analysis is named semiquantitative analysis based also on relative signal intensity of the curve as descriptive analysis. Area under the enhancing curve may be easily calculated and the initial area under the curve (before 60 sec) named Initial Area under Curve (IAUC60) has been demonstrated useful for adnexal masses characterization [28, 29]. Using different mathematic models, the time intensity curve can be fitted. With a high temporal resolution acquisition data, Thomassin-Naggara et al. determined three semiquantitative parameters by fitting with Hill equation: enhancement amplitude (EA), time of half rising (THR), and maximal slope (MS) of the curve. These parameters were demonstrated to be useful to characterize adnexal masses because a correlation was proven between enhancement amplitude and maximal slope with pericyte coverage index (PCI) and high expression of VEGFR-2 on both epithelial and endothelial cells [25]. Moreover, an independent external validation of these parameters was performed later on another population studied in another center [29]. With a lower temporal resolution acquisition data of 30 seconds, Dilks et al. demonstrated the usefulness of other parameters including mean SI_max_, a lower SI_rel_, and a wash-in rate (WIR) that corresponds to the ratio between enhancement amplitude and time [30] (Figure 3). These authors underlined the simplicity of this technique because the software used to calculate enhancement parameters is widely available on MR imaging workstations [31].

**Figure 4:**
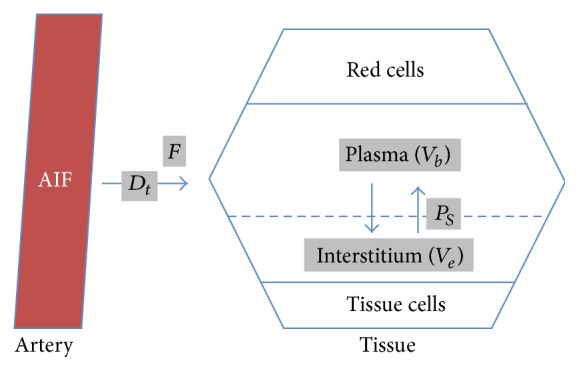
Pharmacokinetic model: Brix modified model with 4 quantitative parameters including tissue blood flow (*F*), blood volumetric fraction (*V*_*b*_), the product of capillary wall permeability and surface area (*P*_*S*_), interstitial volume (*V*_*e*_), and the delay for the contrast media to reach tissue (*D*_*t*_).

As we underlined before, descriptive and semiquantitative analysis are based on signal intensity evaluation. However, using MR imaging, signal intensity depends mainly on the type of acquisition parameters, such as flip angle and TR. Image contrast provided by the administration of contrast agent and linearity between signal intensity variation and contrast agent concentration are highly dependent on these parameters [32]. Thus, many authors argue that we need to obtain reproducible perfusion parameters independent of acquisition conditions. We needed to develop perfusion parameters that are not expressed according to signal intensity but to gadolinium concentration [33]. Then, a more recent approach consists in a quantitative analysis which is based on a pharmacokinetic modeling allowing conversion of signal intensity into gadolinium concentration [34, 35]. Depending on the temporal resolution of the acquisition, different pharmacokinetic models may be applied. At low temporal resolution acquisition (>10 sec), Carter et al. demonstrated the usefulness of Tofts-Kety model to differentiate benign from malignant tumors using *K*_trans_ and *K*_ep_ [29]. Tofts-Kety model is the most used pharmacokinetic model in this context thanks to its good reproducibility [35]. However, the original Tofts-Kety is not a physiological model resulting in parameters with values depending on acquisition settings [36]. Moreover, descriptive and semiquantitative analysis showed that the initial part of the curve is the most informative to distinguish benign from malignant tumors which suggests that tissue blood flow would be the most informative parameter. Thus, using high temporal resolution acquisition (3 sec) [37], de Bazelaire et al. described a Brix modified model [38] that allows the determination of tissue blood flow (*F*_*T*_), blood volume fraction (*V*_*b*_), permeability-surface area product (*P*_*S*_), and interstitial volume fraction (*V*_*e*_) and this proved useful for adnexal mass characterization [39] (Figure 4).

### 2.4. Added Value for Adnexal Mass Characterization

The perfusion weighted MR sequence provides additional criteria for adnexal characterization.

**Figure 5:**
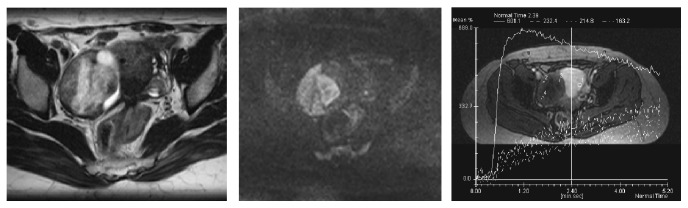
Ovarian fibroma right adnexal mixed cystic solid mass with an intermediate T2 weighted signal intensity in the solid component (A), a high DW signal (B), and a time intensity curve weak and progressive without any plateau (dotted line) in comparison with myometrial enhancement (continuous line).

**Figure 6:**
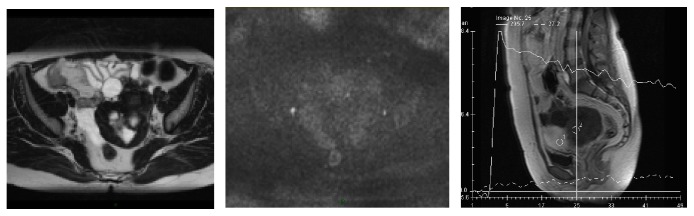
T2 “dark though” effect—cystadenofibroma. The fibrous component of this tumor was highly cellular with an ADC value lower than 1 · 10^−3^ mm^2^/s. However, the tumor is not bright on DW image because of its low T2 signal (T2 dark through effect).

Using descriptive analysis, first publications demonstrated in a population of 37 epithelial tumors that a slow, low-level enhancement pattern typical of a type 1 curve has a sensitivity of 70% and a specificity of 88.8% for benign tumor, whereas a rapid and high level of enhancement typical of a type 3 curve was only found in invasive malignant tumors with a sensitivity of 67%. A type 2 curve was mostly found in borderline tumors (sensitivity 72.4%) but with a lower specificity [28]. The added value of perfusion weighted imaging was tested in a larger population of 87 complex adnexal masses [24] and the addition of time intensity curve analysis resulted in an increase in diagnostic confidence of 25% for a senior reader in pelvic MR imaging. The diagnosis was correctly changed in 7% (3/41) of malignant masses and in 62% (10/16) of benign masses. No diagnosis was incorrectly changed. In our experience, the time intensity curve analysis is particularly interesting for benign tumors with fibrous component such as ovarian fibroma or cystadenofibroma that do not always display a low T2 signal due to oedematous areas. These lesions typically enhance with time intensity curve type 1 [40] (Figure 5). Moreover, time intensity curve analysis is also useful to differentiate borderline from invasive malignant tumors. Typically, borderline tumors display solid papillary projections whereas invasive malignant tumors display a solid mass with or without solid papillary projections. When there is no clear solid mass but grouped solid papillary projection, it can be difficult to be sure if the tumor is only a borderline tumor or an early invasive ovarian cancer. Another application of time intensity curve analysis is to help characterize a solid pelvic mass when the ovarian or uterine origin cannot be clearly identified. In menopausal women, normal ovarian parenchyma is more difficult to identify on T2 weighted sequences than in premenopausal women because follicles are usually no longer seen. The two most frequent solid pelvic masses in women are uterine leiomyoma and ovarian fibroma. When a uterine leiomyoma is pedunculated, morphological criteria are sometimes insufficient to be sure of the origin. Moreover, T2 signal intensity is not very useful to distinguish these two tumors. Time intensity curve analysis can be useful in this situation because uterine leiomyomas are typically enhanced with virtually the same time intensity curve as the myometrium whereas ovarian fibromas display a weak and progressive manner (type 1 curve) [41] (Figure 6).

Using semiquantitative analysis, Bernardin et al. demonstrated that benign lesions displayed a lower mean maximal signal intensity (SI_max_), a lower relative signal intensity (SI_rel_), and a lower wash-in rate (WIR) than borderline and malignant tumors [42] (Figure 3). In this study, the authors found a sensitivity of 67% and specificity of 88% in predicting borderline/invasive malignancy applying a cutoff WIR of 9.5 l/s, although there was overlap between borderline and benign lesion with a range of 2–8 l/s. Moreover, in a more recent study, the same team confirms that all benign cystadenofibroma solid tissues have a WIR less than 5.8 l/s [40]. Using myometrium as internal reference, Thomassin-Naggara et al. showed that the enhancement amplitude ratio (EA_ratio_), time of half rising ratio (THR_ratio_), and maximal slope (MS_ratio_) were significantly higher in invasive malignant tumors than in benign and borderline tumors. Moreover, in this last study, borderline tumors were characterized by a higher EA_ratio_ than benign tumors whereas no differences between benign and borderline tumors were found using WIR values [43]. Thus, in our experience, the use of myometrium as internal reference improves the diagnostic value of semiquantitative parameters when viewed in combination with the descriptive parameters.

Using quantitative analysis, Thomassin-Naggara et al. demonstrated that benign tumors displayed a lower tissue blood flow (*F*_*T*_), a lower blood fraction volume (*V*_*b*_), a higher interstitial volume (*V*_*e*_), and a lower relative AUC (rAUC) than malignant tumors. Moreover, borderline tumors displayed a lower *F*_*T*_ and a higher *D*_*t*_ than invasive ovarian tumors [39]. With data acquired at lower temporal resolution, malignant tumors display a higher *K*_ep_ in their solid component than benign tumors (*P* < 0,001) [29].

## 3. Diffusion Weighted MR Sequence

Diffusion weighted (DW) MR imaging is based on the analysis of the movement of water molecules in a tissue. In a highly cellular tissue, a restriction of the movement of water molecules (a reduction in diffusivity) exists between cells and the tissue appears as high signal intensity on high *b* value DW image with associated low ADC (apparent diffusion coefficient). In less cellular tissue, there is less restriction of the movement of water molecules. Thus, ADC is high and the tissue typically appears without any high signal intensity on the high b value DW images. Indeed, depending on acquisition parameters, DW signal will be due to both cellularity (ADC values) and T2 signal.

### 3.1. MR Acquisition Parameters and MR Analysis

For adnexal mass characterization, many studies have underlined the usefulness of DW signal intensity, although the ADC analysis for solid component analysis can be unhelpful [29, 44, 45]. In fact, many benign tumors have fibrous tissue that restricts the movement of water molecules. Thus, there is a great overlap between ADC values of the solid tissue of benign and malignant tumors [29, 45]. However, as the DW signal is the combination of water diffusivity and T2 signal intensity, the typically very low T2 weighted signal intensity of fibrous tissue in these benign adnexal tumors decreases the theoretical high DW signal intensity due to the high cellularity of these tumors (T2 dark though effect) (Figure 6). Thus, the analysis of DW signal allows correct classification as benign in this group of tumors and makes such criteria accurate to distinguish benign from malignant adnexal tumors.

Then, as mainly DW signal is useful for adnexal mass characterization, we need to optimize a sequence to obtain a sequence with the lowest T2 signal effect with a maximal contrast to noise ratio. T2 signal effect decreases with the increase of *b* values. At *b*_0_ value, the sequence is weighted as a T2 weighted sequence. To find the optimal *b* value, we use urine as internal reference. As we want to suppress T2 effect, we need to obtain dark urine. In our experience, the right *b* value is between *b*_1000_ and *b*_1200_ depending on the manufacturer.

### 3.2. Added Value for Adnexal Masses Characterization

Many publications have studied the value of diffusion MR sequence for adnexal mass characterization. First publications studied the value of diffusion weighted MR imaging to characterize endometriotic cystic component or mature cystic teratoma [46]. The accuracy of MR imaging with conventional sequences is very high for these benign masses (up to 95%) [14, 47–49]. Thus, the added value of diffusion weighted imaging in this setting is limited and has never been statistically demonstrated. Nevertheless, in mature cystic teratoma with paucity of fat, the very low ADC value should be useful to identify keratinoid content [46, 50].

**Figure 7:**
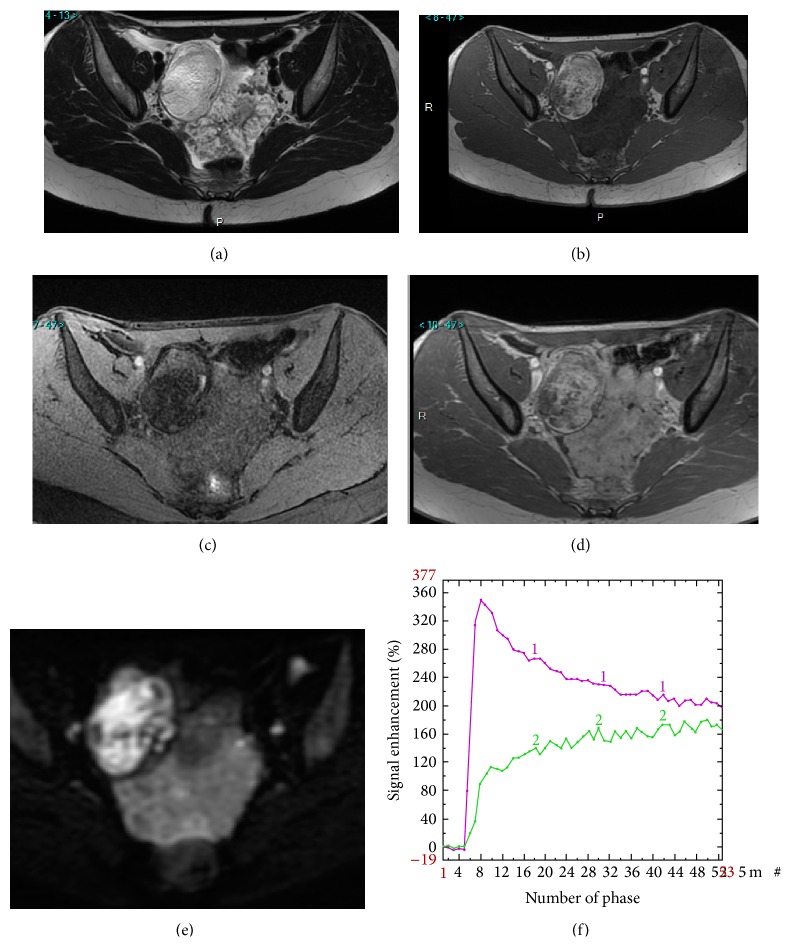
Added value of perfusion and diffusion weighted imaging. Right side: mature cystic teratoma (purely fatty mass: A_DNEX_MR_score_ 2). Left side: borderline serous cystadenoma (solid tissue which is bright on T2 and DW sequence and that enhances with a TIC type 2: A_DNEX_MR_score_ 4). T2 weighted sequence (a), T1 weighted sequence (b), T1 weighted sequence with fat saturation (c), T1 weighted sequence with gadolinium (d), DW sequence (e), and PW analysis comparing myometrial TIC (1) and tumoral TIC (2) (f).

Using DWI to characterize solid tissue, many authors demonstrate that ADC values are not useful to distinguish benign from malignant tumors due to the overlap with benign fibrous tumors [44, 51]. However, thanks to “dark through” effect, the value of the absence of DW signal was demonstrated for predicting benignity (positive likelihood ratio = 10.1). Moreover, the combination of a low T2 weighted signal with a low DW signal allows a confident exclusion of malignancy. These preliminary data were confirmed in further studies that underlined the added value of diffusion weighted sequence to increase diagnostic confidence in 15% and especially for benign tumors [24]. The diagnosis was correctly changed in 8.9% (4/45) of malignant masses and in 28% (8/28) of benign masses. One diagnosis was incorrectly changed in one case of ovarian fibroma (Figure 7).

## 4. Combination of Functional Parameters in an Interpretation Model

Finally, some studies have evaluated the usefulness of the added combination of perfusion and diffusion weighted analysis to conventional analysis. These studies demonstrated that functional analysis is useful especially for reclassifying as benign tumors that were misclassified as malignant using conventional criteria. In our experience, that is the main issue for clinical practice. Indeed, recent trials have shown that unnecessary interventions in women with benign adnexal lesions lead to significant morbidity and mortality [52]. All women have operative and anesthetic risks, which are increased in menopausal women with additional comorbidity factors (obesity, diabetes, and hypertension). Moreover, in premenopausal women, preservation of fertility is a major issue as ovarian surgery mainly based on cystectomy is associated with the risk of ovarian reserve alteration, especially for cysts greater than 5 cm in diameter [53, 54].

Although MRI is the most accurate imaging technique to characterize adnexal masses [54, 55], a debate exists on its use among clinicians explaining why only 25% of surgeons performed MR imaging before surgery and thus the high incidence of preoperative misdiagnoses [23].

**Figure 8:**
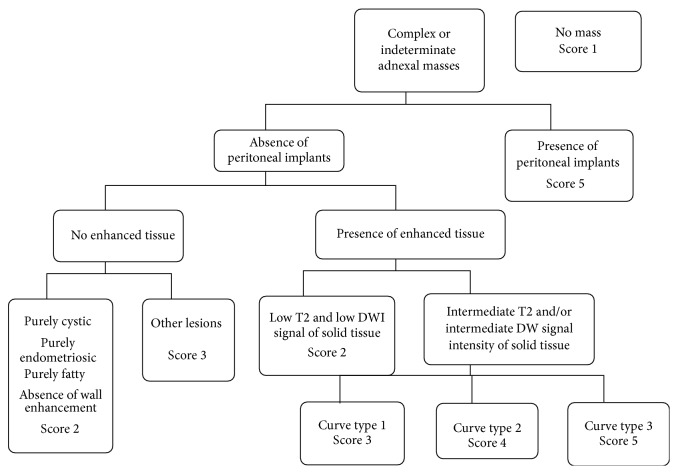
A_DNEX_ MR scoring system (19).

One hypothesis is the lack of standardization of the MR report and the absence of combination of conventional and functional criteria and this could in part be responsible. In this context, the first MR diagnostic score was recently built and described each complex adnexal mass according its positive predictive value of malignancy with a five categories score named A_DNEX_MR_SCORING_ system [19] (Table 2). If the complex mass does not display any enhancing solid tissue, the classification is A_DNEX_MR_SCORE_ 2 or 3 with a PPV lower than 5%. If a complex adnexal mass displays an enhancing solid tissue, in the absence of peritoneal implants, we need to take account of the evaluation of diffusion weighted (DW) signal and time intensity curve analysis. Functional sequences are included in this score as follows: If there is a low T2 and low DW signal on the high *b* value image, the tumor is always benign (A_DNEX_MR_SCORE_ 2). If not, time intensity curve analysis allows differentiating probably benign masses (A_DNEX_MR_SCORE_ 3) (if the solid tissue is enhanced according to a type 1 time intensity curve), indeterminate mass (A_DNEX_MR_SCORE_ 4) (if the solid tissue is enhanced according to a type 2 time intensity), and probably malignant mass (A_DNEX_MR_SCORE_ 5) (if the solid tissue is enhanced with a type 3 time intensity curve) (Figure 8). This score was developed and validated with a very high accuracy (AUROC > 0.94). A score ≤ 3 has been associated with benignity with a sensitivity of 96.6% and a specificity of 93.5%. A multicentre European prospective study (the “EURAD study”) is currently in recruitment, with the aim of validating the proposed scoring system (clinical trial NCT01738789). The potential impact of the score on therapeutic management will then be tested in a future trial.

In this review, we present the development of perfusion and diffusion analysis to characterize sonographically indeterminate adnexal masses. Using time intensity curve analysis and visual assessment of DW signal, functional criteria help the radiologist to improve lesion characterization especially for benign lesions and should help the clinician to avoid unnecessary surgeries. Currently, few data are available to validate in clinical routine more reproducible perfusion analysis for the characterization of adnexal masses. Very recently, new developments of DWI were reported thanks to 3T acquisition including the analysis of the heterogeneity of the tumor (ADC entropy) [51]. This method of research would be interesting in the future to find new criteria of characterization.

## 5. Key Points

(1) Perfusion weighted and diffusion weighted MR imaging help to characterize adnexal masses.
(2) Benign tumors with solid components typically display a low DWI signal.
(3) Benign solid tumors are typically enhanced with a type 1 time intensity curve.
(4) A_DNEX_MR_SCORING_ system is helpful to relay the radiologist's suspicion of malignancy.

## Figures and Tables

**Table 1:** Acquisition parameters.

Sequences	Parameters
Sag T2 without FS	(i) **FOV**: 24 cm (ii) **Thickness** ≤6 mm (iii) +/− **motion correction** if available

Ax T2 without FS	(i) **FOV**: 30 cm, from kidney to symphysis. (ii) **Thickness**: 5 mm/1.0 spacing

Ax T1 Ax or sag T1 FS	(i) **Exact same location as Ax T2 without FS** (ii) **FOV**: 30 cm (a) **2D** thickness 5 mm/1.0 spacing (b) **3D** reconstruction thickness 3 mm/0.0 spacing

Ax DWI	(i) **Exact same location as Ax T2 ** **without** **FS** (ii) *b* **value**: 1000–1200 with black urine (iii) **Thickness**: 6 mm/0.0 spacing or 5 mm/1.0 spacing

Ax PWI	(i) 3D T1 without FS (ii) **Temporal resolution** <15 sec (iii) **Spatial resolution and slice thickness** = 3 mm/0.0 sp (iv) **Size of box** >15 cm (v) **Loc. per slab** >50 (vi) 4 acquisitions without gadolinium (baseline) (vii) **Reformat good quality** of sagittal and coronal reconstruction +++ (viii) **Time duration**: 4 mn

Ax or sag T1 FS gadolinium	(i) Copy Ax or sag T1FS without gadolinium

**Table 2:** A_DNEX_MR_SCORING_ system.

A_DNEX_MR_SCORE _1	Absence of mass	—
A_DNEX_MR_SCORE _2	Benign mass	Purely cystic mass Purely endometriotic mass Purely fatty mass Mass without wall enhancement Low T2 and low DW signal of solid component

A_DNEX_MR_SCORE _3	Probably benign mass	Bi- or multiloculate cyst without solid component Curve type 1 of solid component

A_DNEX_MR_SCORE _4	Indeterminate	All others lesions (including curve type 2 of solid component)

A_DNEX_MR_SCORE _5	Probably malignant mass	Curve type 3 of solid component Peritoneal implants

## Abbreviations

MR: Magnetic resonance
VEGF: Vascular Endothelial Growth Factor
DCE: Dynamic Contrast Enhanced
GRE: Gradient Echo
IAUC: Initial Area under Curve
EA: Enhancement amplitude
THR: Time of half rising
MS: Maximal slope
PCI: Pericyte coverage index
WIR: Wash-in rate
*F*_*T*_: Tissue blood flow
*V*_*b*_: Blood volume fraction
*P*_*S*_: Permeability-surface area product
*V*_*e*_: Interstitial volume fraction
TR: Time of relaxation
SI_max_: Maximum signal intensity
EA_ratio_: Enhancement amplitude ratio
THR_ratio_: Time of half rising ratio
MS_ratio_: Maximal slope
ADC: Apparent diffusion coefficient.
